# Genomic Characterization of a Large Outbreak of *Legionella pneumophila* Serogroup 1 Strains in Quebec City, 2012

**DOI:** 10.1371/journal.pone.0103852

**Published:** 2014-08-08

**Authors:** Simon Lévesque, Pier-Luc Plante, Nilmini Mendis, Philippe Cantin, Geneviève Marchand, Hugues Charest, Frédéric Raymond, Caroline Huot, Isabelle Goupil-Sormany, François Desbiens, Sébastien P. Faucher, Jacques Corbeil, Cécile Tremblay

**Affiliations:** 1 Laboratoire de Santé Publique du Québec (LSPQ)/Institut National de Santé Publique du Québec, Québec, Canada; 2 Université Laval, Department of Molecular Medicine, Québec, Canada; 3 Department of Natural Resource Sciences, Faculty of Agricultural and Environmental Sciences, McGill University, Québec, Canada; 4 Centre d'Expertise en Analyse Environnementale du Québec, Québec, Canada; 5 Institut de Recherche Robert-Sauvé en Santé et en Sécurité du Travail, Québec, Canada; 6 Direction Régionale de Santé Publique de la Capitale-Nationale, Québec, Canada; 7 Département de Microbiologie, Immunologie et Infectiologie, Université de Montréal, Québec, Canada; 8 Centre de Recherche du Centre Hospitalier de l'Université de Montréal, Québec, Canada; University of Louisville, United States of America

## Abstract

During the summer of 2012, a major *Legionella pneumophila* serogroup 1 outbreak occurred in Quebec City, Canada, which caused 182 declared cases of Legionnaire's disease and included 13 fatalities. *Legionella pneumophila* serogroup 1 isolates from 23 patients as well as from 32 cooling towers located in the vicinity of the outbreak were recovered for analysis. In addition, 6 isolates from the 1996 Quebec City outbreak and 4 isolates from patients unrelated to both outbreaks were added to allow comparison. We characterized the isolates using pulsed-field gel electrophoresis, sequence-based typing, and whole genome sequencing. The comparison of patients-isolated strains to cooling tower isolates allowed the identification of the tower that was the source of the outbreak. *Legionella pneumophila* strain Quebec 2012 was identified as a ST-62 by sequence-based typing methodology. Two new *Legionellaceae* plasmids were found only in the epidemic strain. The LVH type IV secretion system was found in the 2012 outbreak isolates but not in the ones from the 1996 outbreak and only in half of the contemporary human isolates. The epidemic strains replicated more efficiently and were more cytotoxic to human macrophages than the environmental strains tested. At least four Icm/Dot effectors in the epidemic strains were absent in the environmental strains suggesting that some effectors could impact the intracellular replication in human macrophages. Sequence-based typing and pulsed-field gel electrophoresis combined with whole genome sequencing allowed the identification and the analysis of the causative strain including its likely environmental source.

## Introduction

During the summer of 2012, Quebec City (Quebec, Canada) experienced one of the largest North American outbreaks of Legionnaires' disease (LD) of the last decades. From July 12 to September 13 2012, 182 cases of LD, including 13 fatalities were reported. *Legionella pneumophila* (*Lp*) has been identified as the most common causative agent of LD, and of Pontiac fever, a milder form of the disease with flu-like symptoms [1]. LD is an acute form of pneumonia that can be quite severe, with a case-fatality rate ranging between 5–30% and approaching 50% in individuals with compromised health status [2]. LD can occur during outbreaks or as sporadic cases, and is an important cause of nosocomial and community-acquired pneumonia (CAP) accounting for 2 to 15% of the latter [3]. *Lp* serogroup 1 (*Lp*1) cause approximately 90% of LD and 95% of community-acquired LD [4], [5].

*Lp* is a gram negative, strictly aerobic bacterium of the *Legionellaceae* family [6]. *Legionella* is found in natural and man-made aquatic environments, such as cooling towers and hot water plumbing infrastructures [2]. Water systems provide optimal growth condition for *Lp* and help its transmission by generating aerosols [7]. Biofilm production is important for persistence of *Lp* in the water system under conditions that preclude its growth [8], [9]. Amoeba have been identified as natural reservoirs for *Lp* in the natural environment and in man-made structures where it infects and replicates inside them [10]–[13].

The majority of LD cases are reported as sporadic cases which can occur throughout the year but mostly in late summer and early autumn [14]. Identifying the source of an outbreak is challenging, as there may be several potential sources in the suspected area. In France, where a surveillance system for *Legionella* is well implemented, environmental sources are identified in only 20% of cases [14], [15]. Furthermore, LD diagnosis presently relies on urinary antigen detection assays which have some limitations and preclude access to primary isolates for typing [16]. Outbreak investigations rely on the rapid identification and typing of the associated organisms in order to identify and control the source of infection [17]. Current standardised typing methods for *Legionella* include monoclonal antibody subgrouping (mAb), pulsed- field gel electrophoresis (PFGE), amplified fragment length polymorphism (AFLP) and sequence-based typing (SBT), which typically take several days [18]. Typing methods directly on the clinical specimens are in development [19], [20] and typing methods directly on environmental water are not available.

At the onset of the Quebec City outbreak, it was suspected that a cooling tower was the putative source. A wide intervention plan was implemented by the public health authorities including environmental sampling and molecular typing of the *Lp* isolates obtained. In order to identify the source of the outbreak, we used classical approaches including PFGE and SBT to type *Lp*1 isolates from both patients and environment. SBT was performed enabling comparison with strains described in an international database. Moreover, we performed whole genome sequencing (WGS) on all patient strains and a selected subset of environmental strains. WGS enabled an in-depth investigation of potential virulence factors and a greater level of certainty as to the identification of the source. WGS represents a valuable approach for tracking and understanding outbreaks.

## Results

### Characterization of *Lp*1 isolates involved in the 2012 Quebec City outbreak

**Figure 1:**
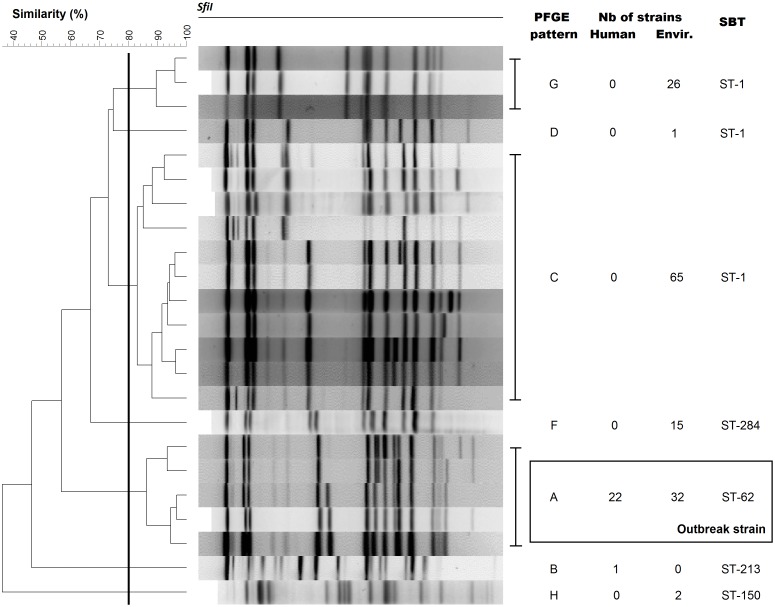
PFGE pattern and SBT types of *Lp*1 isolates involved in the 2012 Québec City outbreak.

*Lp1* isolates recovered from 23 patients (n = 23 isolates) and from 32 (24%) cooling towers (n = 146 isolates) located in the vicinity of the outbreak discriminated into seven different PFGE profiles using *SfiI* (**Figure 1**). From the 23 *Lp* isolates obtained from humans, 22 displayed a unique PFGE cluster (pattern A). The strain from one patient with the different PFGE pattern (pattern B) represented a sporadic case unrelated to the outbreak. Among the 146 *Lp1* isolates analysed from cooling towers, 6 different PFGE patterns (patterns A, C, D, F, G, H) were identified. Only one cooling tower system (two towers linked together) harbored strains with the same PFGE pattern (pattern A) as patients and it was identified as the source of the outbreak. The allelic profile of 53 isolates (23 patients and 30 environment isolates) with a minimum of one strain typed for each PFGE pattern was typed by SBT. The 53 isolates corresponded to 5 different sequence types (STs) (**Figure 1**). All patient isolates and isolates from the cooling tower with PFGE pattern A corresponded to ST-62. The patient with PFGE pattern B was ST-213. ST-1 was the most frequent type obtained from cooling towers (65%). ST-284 and ST-150 were found in 11% and 1.4% cooling towers isolates, respectively.

**Figure 2:**
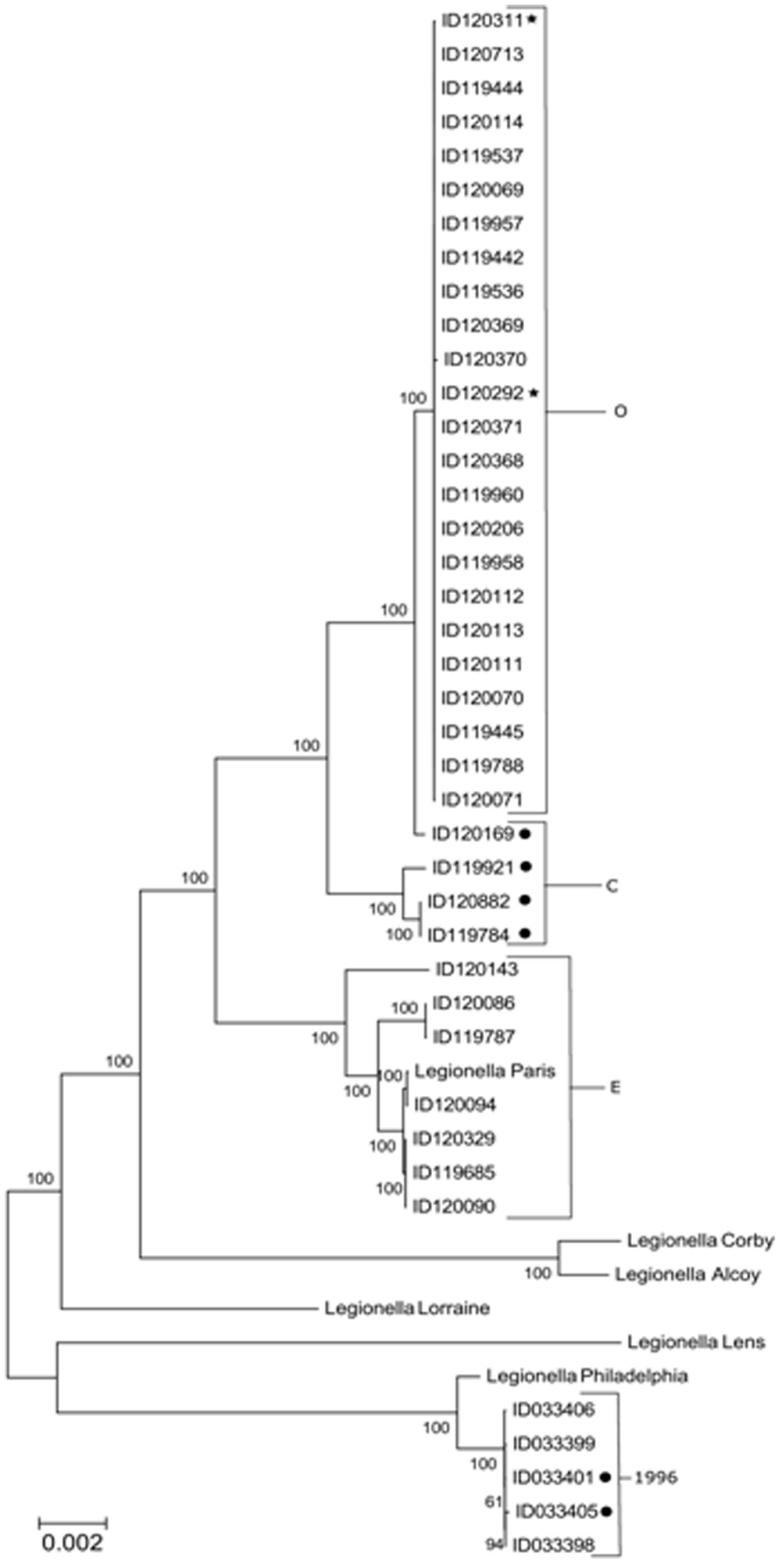
Phylogenic tree of *Legionella pneumophila* strains. There are 4 groups: outbreak related (O), contemporary (C), environmental (E) and 1996 strains (1996). The samples marked with a star symbol are from the cooling tower that was the source of the outbreak. The samples marked with a dot symbol are from patient unrelated to the outbreak of 2012. There is an average of 0.01 base substitution per site overall and an average of 0.0003 base substitution per site for the patient group.

We sequenced with the Illumina technology the genome of 23 *Lp* isolates from patients related to the outbreak, 4 isolates from contemporary patients not related to the outbreak, 8 isolates from cooling towers and 5 *Lp* isolates gathered during the 1996 Quebec City outbreak. Isolates from cooling towers were chosen to represent each environmental PFGE patterns. Whole genome phylogeny of these sequences and reference *Lp* genomes were constructed (**Figure 2**). This phylogeny was constructed based on an alignment of all the contigs of every strain and 6 reference sequences. All outbreak patient strains clustered together in the phylogenetic tree, showing that they are closely related. One of the sampled environmental sites clustered with the patient isolates, indicating that it was the source of the outbreak. Our data showed that the Québec City outbreak strains differed from strains observed in other major outbreaks such as the Philadelphia, Paris, Lens, Corby and Alcoy strains (**Table 1** **and** **Figure 2**).

**Table 1:** Genetic characterization of *Legionella* isolates collected during the 2012 Quebec City outbreak compared to other strains involved in large epidemics.

	Presence in strains studied^a^									
Genetic element	Patient	Source	Environment	Contemporary	1996	Corby	Paris	Lens	Philadelphia	Alcoy
LVH Type IV secretion system	Yes	Yes	Yes	3/6	No	No	Yes	Yes	Yes	No
Type II secretion system	Yes	Yes	Yes	Yes	Yes	Yes	Yes	Yes	Yes	Yes
*lpg1057*	No	No	Yes	No	No	No	Yes	Yes	Yes	No
Plasmid 51.5 and 144 kb	Yes^b^	Yes	No	No	No	No	1^c^	1^d^	No	No

a Patient isolates related to the outbreak (patient), patient isolates obtained in the same period of time from elsewhere in the province of Quebec (contemporary), a subset of environmental isolates with different PFGE pattern than the epidemic strain (environment), isolates from the environmental source of the outbreak (source) and isolates from a former outbreak in the same area in 1996.

b One patient isolate had no plasmid (ID102206) and one patient isolate had only the 51.5 kb plasmid (ID120070).

c A 131.9 kb plasmid was present (plasmid pLPP accession number NC_006365).

d A 59.8 kb plasmid was present (plasmid pLPL accession number NC_006366).

### Genome sequencing of *Lp*

**Figure 3:**
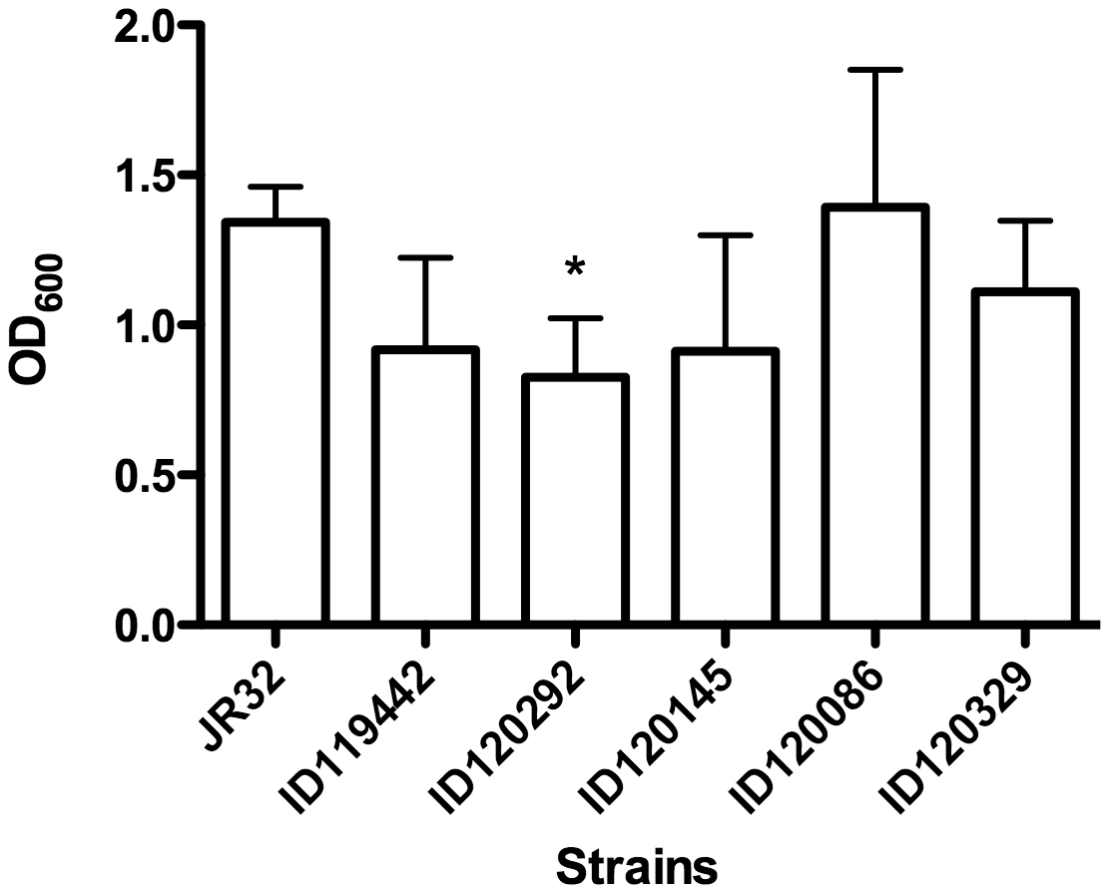
Production of biofilms by *L. pneumophila* serogroup 1 strains. One strain from patient (ID119442), one strain from the outbreak source's cooling tower (ID120292), and three from environment (other that the outbreak source) (ID120145, ID120086 and ID 120329) were tested. Star represent difference statistically significant (*p* = 0.0065) with control strain JR32. Error bars represent standard deviations derived from four independent experiments.

WGS was performed to characterize the strains for putative virulence markers that could have contributed to the major Quebec City *Legionella* outbreak. The epidemic strain harbored a 50.5 kb plasmid comprising 55 genes and a 144 kb plasmid harboring 158 genes. The smaller one had two sections similar to two plasmids from *Fluoribater dumofii* plasmids: pLDNY1 and pLD-TEX-KL. It mainly contained genes for pili formation and conjugation with 25% hypothetical genes. The bigger one was similar to pLELO plasmid from *Legionella pneumophila*. It contained genes associated with biological and metabolic processes. The longest plasmid also harboured genes associated with drug resistance: an erythromycin ABC transporter ATP-binding protein (similar to gene *lpg1616*) and a beta-lactamase AmpS (similar to gene *LPC_1045*). However no clinical resistance to these antibiotics was detected in the patient strains (data not shown). The larger plasmid had nearly 50% hypothetical genes. Type II and Dot/Icm Type IV secretion system were present in all strains. LVH Type IV secretion system was found in all strains except for those unrelated to the outbreak patient strains and 1996 strains. We have identified at least 4 Icm/Dot effectors in the epidemic strain that were absent in environmental strains (**Table 2**), from a list of 140 effectors found in *Lp*1 strains. We also found that gene *lpg1057* was missing in the outbreak strain's genome but was present in environmental strains (**Table 1**). Recently, it was shown that *lpg1057* is linked with biofilm formation [21]. We performed a biofilm formation assay measured by crystal violet staining to confirm that strains lacking *lpg1057* were producing less biofilm than other strains harboring the gene. Only the strain ID120292 from the environmental source had a significantly lower level of biofilm production (*p* = 0.0065) (**Figure 3**).

**Table 2:** Presence of selected Icm/Dot effectors in strains isolated in Quebec City.

Effector	Presence in strains studied^a^			
name	Patient	Environmental	1996	Others clinical
*lpg1120*	Yes	No	Yes	Yes
*lpg2523*	Yes	No	Yes	Yes
*lpg2826*	Yes	No	Yes	Yes
*legC8*	Yes	No	Yes	Yes

a Patient isolates related to the outbreak (patient), a subset of environmental isolates with different PFGE pattern than the epidemic strain (environment), isolates from a former outbreak in the same area in 1996 and other clinical strains (Lens, Corby, Lorraine, Philadelphia-1, Paris and HL06041035).

### Interaction of Québec strains with host cells

**Figure 4:**
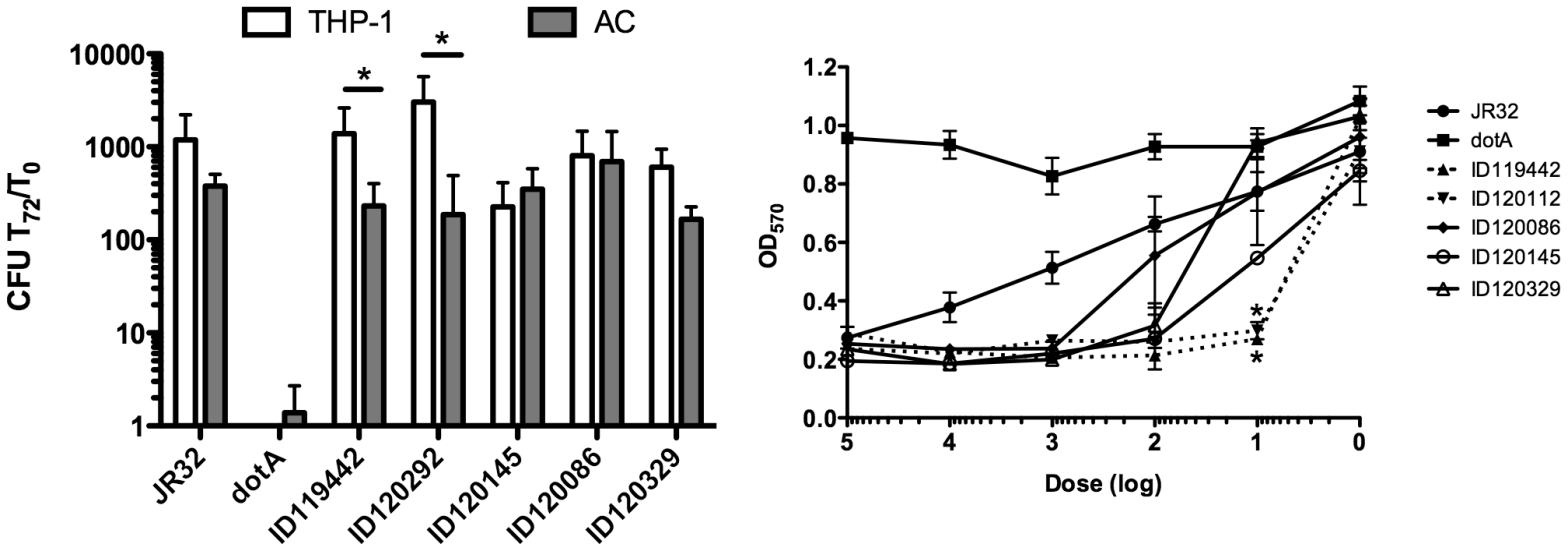
Interaction of *L. pneumophila* serogroup 1 strains with host cells. One strain from patient (ID119442), one strain from the outbreak source's cooling tower (ID120292), and three from environment (other that the outbreak source) (ID120145, ID120086 and ID120329) were tested. JR32 and an isogenic *dotA* deficient strain was used as a negative control for intracellular multiplication (ICM) and cytotoxicity. A) ICM in infected human macrophages (THP-1) and *Acanthamoeba castellanii* (AC). The data represent the ratio between the CFU at time 72 h and the CFU at time 0. Star represent difference statistically significant (*P*<0.01). B) Evaluation of cytotoxicity on the viability of THP-1 cells after infection for 5 days with the MTT assay. The star represents difference statistically significant (*P*<0.01). Error bars represent standard deviations derived from three independent experiments.

We evaluated the intracellular multiplication in human cultured macrophages (THP-1) and in *A. castellanii* (AC) and cytotoxicity toward THP-1 cells. The JR32 strain, derived from Philadelphia-1 and the isogenic Icm/Dot deficient *dotA* mutant was used as a positive and negative control respectively for both assays [22]. As seen in **Figure 4A**, the outbreak strains (clinical, ID119442 and the environmental source, ID120292) replicated more efficiently in human THP-1 macrophages than in AC (*p*<0.05). In contrast, environmental strains were equally capable of replicating in THP-1 cells and AC (ID120145, ID120086 and ID120329). The control strain JR32 seems to grow better in THP-1 than AC, but the difference was not significant. There was no difference of replication in AC between the strains tested. The cytotoxicity toward THP-1 cells was investigated by measuring cell viability with the MTT assay. The dose-response curves revealed that the clinical strains were significantly more cytotoxic (*p*<0.01) than environmental strains toward THP-1 cells at a dose of 10. (**Figure 4B**).

## Discussion

**Figure 5:**
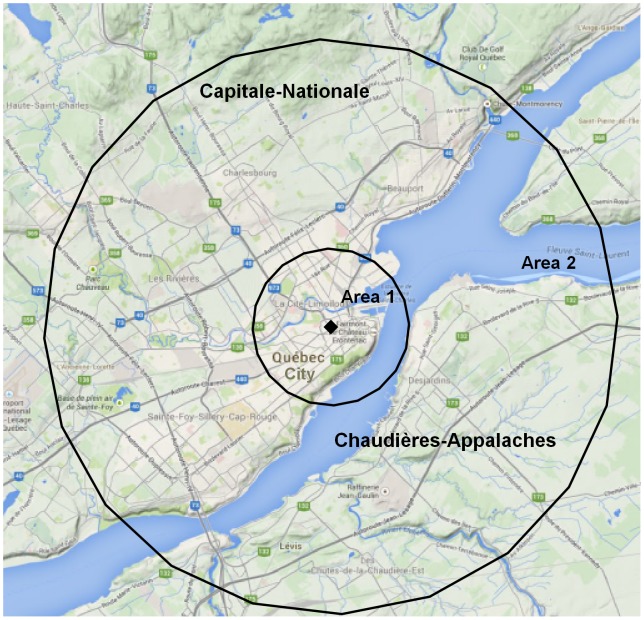
Mapping of the legionellosis cases around the outbreak source. Ninety six percent (96%) of the cases were within 3 km (area 1) of the cooling tower responsible for the outbreak (identified by the black dot). The remaining cases were within 11 km of the outbreak source (area 2).

We report on one of the largest North American outbreaks of LD of the last decades. This outbreak had 182 confirmed cases including 13 fatalities. The LD cases diagnosed spanned a large geographical area, however 96% of the cases were within a 3 km range (Figure 5). Several of the sampled cooling towers contained *Lp*1. Ultimately the source of the epidemic was traced to a single cooling tower. The epidemiological typing scheme of the Quebec City outbreak (ST-62) was already described in North America and Europe according to the SBT international database. In Canada, ST-62 had been identified in Ontario, Nova-Scotia and on one occasion in Quebec City in 1998 [23], [24]. ST-213 was identified for one patient considered a sporadic case. This type is rarely reported and is restricted to North America according to the SBT database. Among *Lp*1 isolates found in the environment, ST-1 was the predominant genotype. ST-1 is one of the most frequent types reported in the SBT database. It was also frequently observed from environment strains in Ontario [24]. This clone is probably well-adapted to environment survival or host infection [24]–[26]. The two other types obtained from the environment, ST-150 and ST-264 are also rare. The first one has been reported from environment in China and Japan, and the second in the USA.

Whole genome sequencing confirmed and validated the findings of PFGE and SBT confirming that only one cooling tower was the source of the outbreak. It also demonstrated that the epidemic strain is genetically distinct from other sporadic *Lp*1 cases in the same period of time, as well as strains from a previous outbreak in the same area and from the five sequenced reference strains. WGS has been previously used for outbreak investigation, either in a nosocomial or community outbreak context [17], [27]. Only one study used WGS to investigate a *Legionella* outbreak in a retrospective setting [17]. Our study represents the largest *Legionella* outbreak investigation using WGS and the first using it in real time. The speed, accuracy, and depth of information provided by WGS platforms make it a method of choice to confirm or refute outbreaks in hospitals and in the community. This approach can also provide information on potential virulence and drug resistance. It can also assist in defining transmission patterns [17]. When combined with traditional epidemiologic investigation, whole-genome sequencing has proven useful for elucidating the sources and transmission dynamics of disease outbreaks.

Sequenced *Lp*1 genomes were analyzed to identify putative virulence markers that could have contributed to the major Quebec City *Legionella* outbreak. The epidemic strain was the only one harboring the LVH type IV secretion system, lacking the *lpg1057* gene and possessing two plasmids.

The most important genetic determinant of intracellular growth is the Icm/Dot type IVb secretion system that injects effectors into the cytoplasm of host cells. These effectors efficiently highjack host cell functions to the bacteria's benefit, allowing them to grow in mammalian macrophages [28]. To date, more than 300 Icm/Dot effectors have been experimentally confirmed [29]–[31]. Genomic comparison of clinical strains of *Lp*1, including Lens, Corby, Lorraine, Philadelphia-1, Paris and HL06041035 [32] revealed that the repertoire of the Icm/Dot effectors possessed by specific strains is variable but that some effectors are highly conserved [33]. The outbreak strains replicated more efficiently in human macrophages than in amoeba and were more cytotoxic to human macrophages than the environmental strains. The genetic cause of this difference is currently unknown. At least four Icm/Dot effectors in the epidemic strains were absent in environmental strains suggesting that some effectors are critical for intracellular replication in human macrophages.

The epidemic strain was the only one lacking *lpg1057*, which is involved in the regulation of biofilm formation and flagellin expression [21], through H-NOX regulation of c-di-GMP metabolism and biofilm formation in *Legionella pneumophila*. Cyclic diguanylate signaling proteins control intracellular growth of *Legionella pneumophila* [34]. No clear association between the presence of *lpg1057* and the level of biofilm production could be drawn.

The two plasmids harbored by the epidemic strain remains to be investigated, since many hypothetical genes were identified. All together with these two plasmids, the epidemic *Legionella* strain acquired nearly 7% new genes demonstrating the extensive plasticity of the species [35]. The role of these plasmids in the virulence of the strain and interaction with the chromosome of the strains remains to be elucidated.

Genotyping strains of *Lp* is a key point to help identify contamination sources. As *Lp* is quite common in the environment, several typing methods have been developed in an attempt to differentiate strains [4]. PFGE is considered as the gold standard method for bacterial typing including the genotyping of *Lp* [18]. However, it is time consuming and comparison between laboratories is difficult. SBT is a powerful method based on the sequencing of seven gene loci [36], [37]. It is now considered the new EWGLI gold standard tool for *Lp* typing and a large sequence types (STs) database is available. Typing results of both typing methods were concordant at identifying the source of the outbreak. Previous study has determined that the PFGE method possessed a high discriminatory power for *Legionella* [38]. Our results show that in the Quebec City outbreak, PFGE was more discriminating that SBT, since more than one PFGE pattern corresponds to only one SBT type (Figure 1). Ultimately, WGS will become the standard since unequivocal comparison can be made between potential source and patient *Lp* sequences as we have demonstrated in this study.

## Conclusion

The Quebec City outbreak was one of the most significant in North America. The use of WGS in conjunction with PFGE and SBT allowed the identification of the source of contamination and an in-depth analysis of the outbreak strain. It is a matter of public health concern to track these outbreaks carefully given the propensity of the *Legionella* species to acquire genes readily. Some of these acquisitions could have serious consequences for the treatment or spread of the disease (resistance to antibiotics or increase in virulence). Further studies are needed to elucidate the functional genetics of *Legionella*. Metagenomic approaches should also be considered in the future to survey rapidly and easily circulating strains in our environment.

## Materials and Methods

### Patient population

In the province of Quebec, the Public Health Director can proceed according to law to investigate an outbreak using patient information, clinical specimens and isolates from patients without the written informed consent of patients. Analysis of clinical specimens and isolates in outbreak investigation context is also the mandate of the Laboratoire de santé publique du Québec (LSPQ). In this context, we obtained samples, collected by hospital microbiology laboratories, from 23 of 183 individuals diagnosed with LD who met the following criteria: i) a person for whom the presence of symptoms started during the outbreak ii) had a radiologically or clinically confirmed pneumonia, and iii) lived within 11 kilometers of the outbreak epicenter or traveled frequently to the area within the 14 days before the beginning of illness. Moreover, the patient specimen had to be positive for one of the following: a culture or urine antigen test or positive immunofluorescence antigen test from respiratory specimens, lung tissue, pleural fluid or positive immunofluorescence antibody serology; IgG titre of ≥1∶128. Isolates which were recovered from clinical specimens were sent to the LSPQ for identification and typing. All data were anomymized prior to analysis and confidentiality was respected under the Public Health and LSPQ mandate.

### Cooling towers culture for *Legionella*

According to the law in an outbreak investigation context, the Public Health Director mandated a team of building inspectors and industrial hygienists to visit all identified buildings that housed cooling tower and to collect water samples. All building owners were informed about the visit. Water samples from cooling towers located near outbreak cases were cultured (131 cooling tower samples from 70 buildings) according to a standardized AFNOR NF T90-431 method as described above [39]. Colonies growing only on BCYE agar were considered as *Legionella* spp. Colonies were then submitted to an agglutination test (Oxoid) to identify *Legionella pneumophila* (serogroups 1 and 2–14). Each isolates of *L. pneumophila* serogroup 1 obtained was sent to LSPQ for confirmation and typing.

### Isolate identification and serogrouping

*Legionella pneumophila* strains were confirmed by indirect fluorescent antibody (IFA) assay (Monofluo *L. pneumophila* kit, Biorad, Canada) at LSPQ. Serogroup 1 strains were ascertained by agglutination test slide (*L. pneumophila* antisera, Denka Seiken, Japan).

### PFGE

All isolates were typed by PFGE. Colonies from a 48 h growth on BCYE plate were harvested in cellular suspension buffer (CSB) (100 mM Tris pH 8.0, 100 mM EDTA pH 8.0) to reach an optical density of 0.48–0.52. To 200 µl of the suspension, 10 µl of proteinase K (20 mg/ml) was mixed. Then, 200 µL of melted 1% SeaKem Gold agarose (Lonza, Basel, Switzerland) was added and the agarose mixture was transferred into a well of the plug mold. The solidified plug was transferred into 270 µL of lysis buffer (50 mM Tris pH 8.0, 50 mM EDTA pH 8.0, 1% sarkosyl) and 30 µl of proteinase K and incubated at 55°C for 1 h with agitation. The plug was rinsed one time with 7.5 ml of purified water at 55°C and then washed two times for 10 min with 7.5 ml of purified water at 55°C and six times for 10 min with 7.5 ml of 1× TE buffer at 55°C (10 mM Tris-HCl pH 8.0, 0.1 mM EDTA). A plug slice of 1 or 2 mm was incubated at 50°C for 10 min in 150 µl of 1× digestion enzyme buffer. After discarding the buffer, 150 µL of a 80 U of *SfiI* solution (New England Biolab, Canada) was added and the plug slice was incubated at 50°C for a minimum of 2 h. The digested slice was loaded in a 1% SeaKem Gold PFGE agarose gel. The Salmonella serotype Braenderup strain (H9812) was used as the size marker in each gel. Electrophoresis was performed using switch times of 5.3 to 34.9 seconds for 19.5 h at 6 V/cm in 0.5× TBE buffer (0.89M Tris pH 8.4, 0.89M boric acid, 0.02M EDTA) at 14°C. Gel was stained in 1× GelRed (Cedarlane, Canada) for 30 min. Gel was visualized with UV light and the picture was digitalized with a Gel Doc XR. The *SfiI* patterns were interpreted using the Tenover's standards [40]. Band position tolerances of 0.8% and optimization value of 1% were used for all analyses. Similarity coefficient was obtained within BioNumerics version 6.5 by calculating Dice coefficients. Cluster analysis was done with the unweighted pair group method with arithmetic averages (UPGMA) using 80% of similarity to assign cluster. PFGE patterns belonging to the same cluster were considered as related and from the same pattern.

### Sequence-based typing

A minimum of 1 strain of each PFGE pattern was typed by Sequence-based typing, according to the European Working Group for *Legionella* infections (EWGLI) protocol [36], [37]. Sequences obtained by Sanger sequencing were analyzed with BioNumerics and compared to the EWGLI database for assigning the sequence type (ST). The obtained STs were submitted to the EWGLI SBT-database (http://www.hpa-bioinformatics.org.uk/legionella/legionella_sbt/php/sbt_homepage.php).

### Whole-genome sequencing

WGS was performed on all patient isolates related to the outbreak, patient isolates obtained in the same period of time from elsewhere in the province of Quebec (contemporary), a subset of environmental isolates, as well as isolates from a former outbreak in the same area in 1996 [41]. WGS was performed using the Nextera XT kit (Illumina, San Diego, CA) as by the manufacturer and the sequencing (2×250 nt) performed on a MiSeq system (Illumina). Mate pair sequencing was performed on the same system using Nextera Mate Pair kit (Illumina) and the associated gel-plus protocol. Sequence assembly was performed using the Ray assembler version 2.2.0 [42]. Reads for the sample corresponding to the best assembly of *Legionella pneumophila* strain Quebec 2012 can be found under project number PRJEB5317 (Sequence Read Archive hosted by EMBL-EBI) and contigs for the same sample are available under accession numbers CCAA010000001 to CCAA010000048.

### Bioinformatics analysis

Prior to construction of the phylogeny tree, alignment of assembled contigs was performed using Mugsy 1.2.3 [43]. Reference sequences from other *Lp* outbreaks around the globe were added to the alignment (Paris (CR628336) [44], Alcoy (CP001828) [45], Corby (CP000675) [46], Lorraine (FQ958210) [32], Lens (CR628337) [44] and Philadelphia (AE017354) [47]). Alignment was converted to fasta, inserting gaps for the sequences where there was no alignment. The phylogeny tree was calculated with MEGA (5.2) [48] using the neighbor joining method and the maximum likelihood substitution model with 500 bootstrap replications. The presence of genes and virulence factors was assessed using BLAST and the virulence factor database (VFDB) [49].

### Biofilm formation

Biofilm formation was measured by crystal violet staining [21]. Strains grown on BCYE plates were suspended in AYE broth (ACES-buffered yeast extract broth supplemented with 0.25 mg/ml ferric pyrophosphate and 0.4 mg/ml L-cysteine) at an OD_600_ of 0.1 after which 200 µl of the bacterial suspension was distributed in triplicate into the middle wells of a 96 well, flat bottom microplate. A triplicate of AYE without bacterial culture was used as a blank. The surrounding wells were filled with 200 µl of distilled water to prevent evaporation of the samples. The microplate was incubated at 30°C for 7 days. At the end of the incubation period, media was removed and the wells were gently washed 3 times with 200 µl of AYE. Wells were then stained with 200 µl of crystal violet (DIFCO) for 10 minutes. The dye was removed and wells gently washed 3 times with distilled water. Finally, 200 µl of 100% ethanol was added to each well and vigorous pipetting removed the crystal violet trapped in the biofilm into solution. The optical density of the wells was measured using a Tecan InfinityPro2000 at 600 nm. The strain JR32 was used as positive control [22]. Mann-Whitney test was used to calculate difference of biofilm production between strains using a significance level of 0.01.

### Intracellular replication

Intracellular multiplication was measured in the amoeba *Acanthamoeba castellanii* and Thp1-derived human macrophages with a multiplicity of infection (MOI) of 0.1, as previously described [50]. Thp1 monocytes were cultured in RPMI (GIBCO) supplemented with L-glutamine and 25% heat-inactivated fetal bovine serum. For the Thp1 infection, cells treated with 10^−7^ nmol phorbol myristate acetate (PMA) were seeded into a 24-well plaque in 1 ml of RPMI, each well containing 5×10^5^ cells 3 days prior to infection and left to incubate at 37°C in 5% CO_2_. One hour prior to infecting the macrophages with *L. pneumophila*, the media in each well was replaced with fresh RPMI. For the amoebal infection, *A. castellanii* were cultured in peptone yeast glucose (PYG) broth (20 g proteose peptone, 1 g yeast extract, 0.1M glucose, 0.4 mM MgSO_4_, 0.05 mM CaCl_2_, 0.1 mM sodium citrate, 0.005 mM Fe(NH_4_)_2_(SO_4_)_2_, 0.25 mM Na_2_HPO_4_ and 0.25 mM KH_2_PO_4_, adjusted pH to 6.5 with HCl). One day prior to infection, 2.5×10^5^ cells in 1 ml of PYG were seeded into each well of a 24-well plaque. Prior to infecting the amoebae with *L. pneumophila*, the media in each well was replaced with 1 ml of AC buffer (PYG medium without proteose peptone, yeast extract and glucose). The laboratory wild type JR32, a streptomycin resistant, restriction negative mutant of the *L. pneumophila* strain Philadelphia-1, was used as a positive control, while a *dotA* mutant of the JR32 strain deficient in intracellular replication served as a negative control [22]. Strains grown on BCYE agar were suspended in AYE broth at an OD_600_ of 0.1 then further diluted 10 fold to obtain an approximate OD_600_ of 0.01. 2 µl of this final solution was used to infect macrophages and amoeba. CFU counts at 24 h intervals were performed to track growth of the bacteria inside the host cells. Mann-Whitney test was used to calculate difference of intracellular replication between strains using a significance level of 0.01.

### Cytotoxicity assay

Thp1 monocytes were cultured in RPMI supplemented with L-glutamine and 25% FBS as described above. The cytotoxicity assay was performed as described by Marra *et al.* [51] Cells treated with 10^−7^ nmol PMA were seeded into a flat bottom 96-well microplate, each well containing 4×10^5^ cells in a total volume of 200 µl 3 days prior to infection and left to incubate at 37°C in 5% CO_2_. Strains were suspended to an OD_600_ of 1 and 10 µl (approximately 10^7^ bacteria) were added to the first row of the microplate in duplicate. A set of duplicate wells was used with 10 µl of AYE containing no bacterial inoculum to serve as a negative control. Then, 10-fold dilutions were made up to a 10^−7^ dilution for each strain. The plate was incubated at 37°C in 5% CO_2_ for 1 week. After the incubation period, 20 µl of 5 mg/ml 1-(4,5-dimethylthiazol-2-yl)-3,5-diphenylformazan (MTT) in PBS was added to each well and the plate incubated for an additional 4 h at 37°C in 5% CO_2_. The supernatant was gently removed and the dark blue formazan precipitate was solubilized using 100 µl of acidified isopropanol (isopropanol with 40 mM HCl). Finally, 10 µl of 20% SDS was added to dissolve the remaining precipitate formed by residual serum and mixed by pipetting. The optical densities of the wells were read at 570 nm using a Tecan InfinityPro2000. Student's t-test was used to calculate difference of cytotoxicity between strains using a significance level of 0.01.
